# Can We Help Care Providers Communicate More Effectively With Persons Having Dementia Living in Long-Term Care Homes?

**DOI:** 10.1177/1533317516680899

**Published:** 2016-11-30

**Authors:** Katherine S. McGilton, Elizabeth Rochon, Souraya Sidani, Alexander Shaw, Boaz M. Ben-David, Marianne Saragosa, Veronique M. Boscart, Rozanne Wilson, Karmit K. Galimidi-Epstein, M. Kathleen Pichora-Fuller

**Affiliations:** 1 Toronto Rehabilitation Institute – University Health Network, Toronto, Ontario, Canada; 2 Lawrence S. Bloomberg Faculty of Nursing, University of Toronto, Toronto, Ontario, Canada; 3 Faculty of Medicine, Department of Speech-Language Pathology, University of Toronto, Toronto, Ontario, Canada; 4 School of Nursing, Ryerson University, Toronto, Ontario, Canada; 5 School of English and Liberal Studies, Seneca College Newnham Campus, Toronto, Ontario, Canada; 6 Communication, Aging and Neuropsychology Lab (CANlab), Baruch Ivcher School of Psychology, Interdisciplinary Center (IDC) Herzliya, Herzliya, Israel; 7 Rehabilitation Sciences Institute (RSI), University of Toronto, Toronto, Ontario, Canada; 8 St Michael’s Hospital, Toronto, Ontario, Canada; 9 School of Health & Life Sciences and Community Services, Conestoga College Institute of Technology and Advanced Learning, Kitchener, Ontario, Canada; 10 School of Nursing, Trinity Western University, Langley, British Columbia, Canada; 11 Centre for Health Evaluation & Outcome Sciences (CHÉOS), University of British Columbia, Vancouver, British Columbia, Canada; 12 Patient-Centred Performance Measurement & Improvement, Providence Health Care, Vancouver, British Columbia, Canada; 13 College of Audiologists and Speech-Language Pathologists of Ontario, Toronto, Ontario, Canada; 14 Department of Psychology, University of Toronto, Mississauga, Ontario, Canada

**Keywords:** resident-centered care, tailored interventions, communication intervention, long-term care homes, staff–resident interactions

## Abstract

**Background:**

Effective communication between residents with dementia and care providers in long-term care homes (LTCHs) is essential to resident-centered care.

**Purpose:**

To determine the effects of a communication intervention on residents’ quality of life (QOL) and care, as well as care providers’ perceived knowledge, mood, and burden.

**Method:**

The intervention included (1) individualized communication plans, (2) a dementia care workshop, and (3) a care provider support system. Pre- and postintervention scores were compared to evaluate the effects of the intervention. A total of 12 residents and 20 care providers in an LTCH participated in the feasibility study.

**Results:**

The rate of care providers’ adherence to the communication plans was 91%. Postintervention, residents experienced a significant increase in overall QOL. Care providers had significant improvement in mood and perceived reduced burden.

**Conclusion:**

The results suggest that the communication intervention demonstrates preliminary evidence of positive effects on residents’ QOL and care providers’ mood and burden.

## Introduction

Dementia is a condition that impairs the cognitive brain functions of memory, language, perception, and thought.^1^ As a result, persons with dementia, especially in the later stages of the disease, may be unable to understand explanations, follow directions, report symptoms, express needs, ask for help, or correctly interpret emotions in verbal communications.^2^ These communication problems have profound implications for effective interactions in long-term care homes (LTCHs). When residents cannot articulate their needs or cannot understand others, behavioral problems, like agitation or depression, may arise. A key component of quality care is the ability of care providers to communicate with residents to understand their needs.^3,4^ In LTCHs, the responsibility for ensuring that a resident receives optimal care often falls on unregulated care providers (hereafter referred to as care providers), such as nursing assistants who may not be equipped with effective communication skills for caring for residents who have communication problems.^5^

In the last decade, the quality of care delivered to LTCH residents has come under scrutiny.^6^ Concerns related to quality of care are further heightened by the increasing complexity of residents’ needs. Almost half (43.8%) of residents are identified as having intermediate to high dependence for activities of daily living (ADL), and those with higher needs (41.5%) tend to be more cognitively impaired.^7^ As well, sensory loss is widespread among older adults and is often overlooked in those living with dementia in residential settings. Approximately 80% of LTCH residents experience hearing loss and approximately 50% of these have a moderate-to-severe impairment.^8^ The prevalence of visual impairment has been reported to be between 30% and 57% in LTCH.^9^ These sensory impairments compound the communication difficulties of residents living with dementia.

Evidence from 4 systematic reviews points to positive outcomes for both residents and care providers when staff receive communication skills training, with limited evidence that residents’ neuropsychiatric symptoms can be influenced.^10-13^ In their review of 19 intervention studies, Vasse and colleagues^10^ found that care providers improved their communication skills when single-task communication strategies (eg, life review, one-to-one conversations) are embedded in daily care activities; however, implementation of these strategies was not associated with changes in residents’ agitation. Another review^11^ examined the effectiveness of communication skills training for care providers in LTCH in 12 trials; the training consisted of didactic methods such as lectures, hands-on training, group discussions, and role-play and was found to improve the quality of life (QOL) and well-being of people with dementia and increase interactions between staff and residents. A third review^12^ focused on methods to enhance verbal communication between residents with dementia and informal and formal care providers. The results of 6 studies indicated that 1 technique (use of memory aids to enhance topic maintenance) along with specific training programs was potentially effective. Following participation in the training programs, care providers reported improvement in knowledge and skills; in particular, they showed increases in their knowledge about dementia and how to respond appropriately to support communication for residents in everyday situations.^12^ The last systematic review focused on identifying the theoretical grounding, components, duration, mode of delivery, and outcomes of communication interventions for health-care providers. In the 6 studies that met the inclusion criteria, the most commonly used components were (1) cognitive, aiming to teach staff about principles and methods of communication, (2) behavioral, focusing on practice of the communication skills at the bedside, and (3) psychological, involving individualized feedback to enhance care providers’ performance of the communication skills in practice.^13^ The authors recommended that to enhance communication skills of care providers, the intervention should be multilevel and comprise 3 components—educational training, practice, and support. In a recent study focused on a communication skills training program,^14^ 24 nursing assistants (NAs) were individually instructed in communication strategies with residents; the instructions were tailored according to each NAs baseline knowledge. After teaching them to use short instructions, positive speech, and biographical statements, and providing feedback in class, the NAs reported less job stress; however, residents did not experience changes in their level of agitation.

In sum, the literature review provides evidence of the effectiveness of the following components in improving care providers’ knowledge and skills in communicating with residents in LTCHs: training in how to use one-to-one communication strategies that are useful in daily care and selecting topics of interest to the resident to engage them in conversation, incorporating didactic methods to instruct staff in the application of new skills, taking the learners’ (ie, care providers) needs into account, and developing a multifactorial intervention focused on education, practice, and support.^10-14^ Nevertheless, this work largely overlooks the possible benefit of tailoring communication strategies that can be applied by care providers to support the individual abilities of the person living with dementia. Current evidence on person-centered care suggests that delivering individually tailored communication interventions may improve the QOL and care of residents with dementia^10^ and those who care for them.

The current novel study addressed this gap by focusing on tailored communication strategies based on the residents’ linguistic, cognitive, and sensory abilities. The study aimed to examine the effectiveness of individualized communication plans tailored to the needs of residents with dementia. Building on our team’s previous research and adapting the Aphasia Framework for Outcome Measurement,^15^ we designed a multifaceted resident-centered communication intervention (RCCI) for people with dementia, which was similar to an intervention we had developed for persons post stroke.^16^ The framework that guided the development of the intervention suggests that 4 domains (participation in life, communication environment, severity of communication disorders, and personal characteristics) can be targeted to enhance the communication environment. The RCCI involved 3 elements that were consistent with the framework propositions: (1) individualized communication plans: the plans incorporated communication strategies that care providers could implement to enhance resident-centered interactions (the strategies were tailored to individual residents’ personal characteristics and the severity of the individuals’ communication disorders), (2) a workshop delivered to care providers to inform them of the principles of communication and to instruct them in the application of the communication strategies with residents (the workshop targeted the communication environment), and (3) a support system for care providers to support the transfer of the newly learned strategies into practice by mentoring staff, which assisted with encouraging the resident to participate in everyday life.

## Research Questions

The present study addressed the following questions:

1. How much do care providers adhere to communication plans developed as part of the RCCI when providing care to residents with dementia?
2. Does the implementation of an RCCI affect care providers’ attitudes toward residents with dementia?
3. Does the implementation of an RCCI affect residents’ depression, ADLs, and QOL?
4. What are the factors that facilitate or impede care providers’ implementation of the communication plans?

## Methods

### Design

A single-group pre versus posttest design was used to examine the effects of the RCCI on resident and care provider outcomes. Recruitment of participants took place between March and November 2014, following approval by the research ethics board.

The implementation of the RCCI was informed by the Promoting Action Research Implementation in Health Services Knowledge Translation framework.^17^ Detailed attention was paid to 3 essential components for successful implementation of the intervention—context, evidence, and facilitation. First, the context included a facility that values resident-centered care and the professional development of staff to maintain high-quality care. The facility administrator supported the focus of the study on individualized communication plans and appreciated the need for enhancing interactions between residents and staff. Second, the evidence was reflected in training care providers in the application of evidence-based communication strategies and instructing them how to tailor the strategies to the abilities of each resident taking into account the learners’ previous experiences and abilities. Third, a facilitator from the study team (MV) acted as a resource to assist the staff in implementing the strategies into practice.

Data on residents’ outcomes were obtained at 2 points in time—before and after care providers implemented the individualized communication plans. For residents’ outcomes, measures of mood and daily functioning were administered at baseline (time 1) and 10 weeks after the care providers were instructed in using the communication plans (time 2). Measures of care providers’ attitudes, satisfaction, and burden were completed at baseline immediately after attending the workshop (time 1) and 10 weeks following the workshop (time 2). Care providers’ adherence to the communication plans was evaluated at time 2. In keeping with recommendations for follow- up after delivering communication programs, after time 2 data collection, focus group sessions were held to explore the barriers and challenges that care providers experienced when implementing the communication plans.^18^

### Setting and Participants

The study took place in a 128-bed, for-profit LTCH, located in an urban center. The RCCI was implemented in the entire facility. Residents were eligible if they (1) had a confirmed diagnosis of dementia, (2) exhibited difficulty being understood by others as assessed by their usual care provider, (3) were assigned to a care provider who was participating in the study, and (4) were able to understand English sufficiently to participate in the study. Residents who were in palliative care were excluded. To protect resident privacy, a facility staff member reviewed residents’ records to determine their eligibility and made the first contact with prospective participants to seek their agreement for the research assistant (RA) to speak to them about the study. Upon agreement, the staff member shared the prospective participants’ contact information with the RA who then approached the participants and/or their proxy to obtain written, informed consent.

Once the residents were recruited, the RA identified the staff responsible for providing their care and invited them to participate in the study. Care providers were eligible if they: (1) provided direct care to a resident participating in the study, (2) worked at least 15 hours per week with the selected resident, and (3) provided written consent.

Of those who were eligible for the study, 12 residents and 20 care providers (10 who worked day shift and 10 who worked evening shift) enrolled. A day and an evening care provider were matched with each participating resident. There were 24 resident–care provider dyads at time 1 but only 22 dyads at time 2 because of the withdrawal of 2 care providers (1 went on maternity leave and the other left employment at the LTCH). Thus, each resident’s outcomes were assessed twice at each time point, except for the 2 residents whose care providers withdrew from the study. The attrition rate for care providers was 8.4%; none of the residents withdrew from the study. Earlier work by the team indicated that a sample size of 18 care providers would provide sufficient power to detect moderate size changes in outcomes in pre- to postintervention comparisons^16^; therefore, the sample sizes analyzed in the present study were considered to be adequate.^19^

### Description and Implementation of the RCCI

The principal investigator (PI) and RA coordinated all components of this multifactorial intervention. The RCCI was implemented over a 10-week period in the following sequence:

#### The development of individualized communication plans by a speech-language pathologist

**Figure 1.**
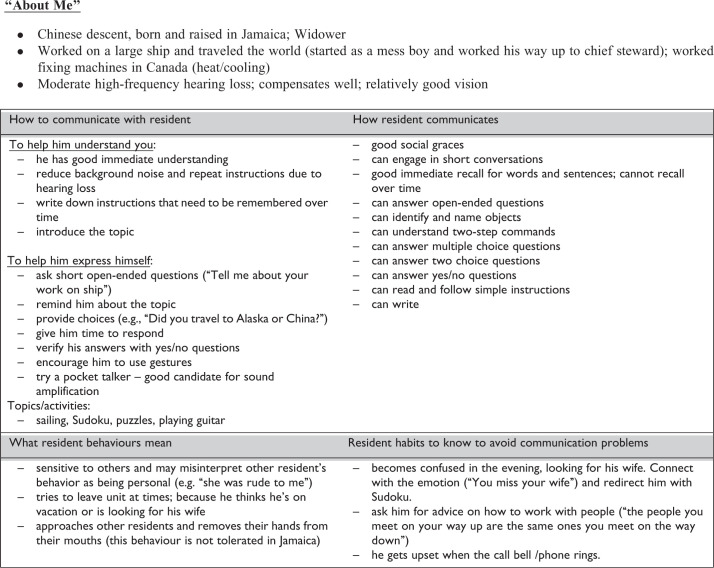
Example communication plan.

The development of the communication plans started with the assessments of vision, hearing, cognition, and functional communication (see below). Next, the information was integrated and informed the generation of an individualized communication care plan. An example care plan is shown in Figure 1. Care plans included how to communicate to the resident, how the resident communicates to others, and an explanation of the resident’s behaviors and habits, including how to avoid communication problems. Information called “about me” also described personal information about the individual, including their hearing and vision abilities.

#### Dementia care workshop including sharing of the communication plans

A 4-hour workshop was developed and delivered to all participating care providers. Research funds were used to cover the cost of staff time to attend the half-day workshop and the cost of replacement staff to cover the shifts missed during the workshop. The workshop was presented on 2 separate occasions so that all care providers could attend. It was delivered by 3 of the authors (K.K.G.E. who is a speech-language pathologist [SLP], K.S.M. and M.S. who are nurses). The aim of the workshop was to discuss communication strategies for use with residents and to gather care providers’ input on the communication plans. The methods used in the workshop included the following:

1. The Relate well, Environmental manipulation, Abilities-focused care and Personhood (REAP) model, developed and previously piloted by the first author,^20^ was used as a guide to teach care providers about strategies that are useful when working with persons having dementia who may have responsive behaviors.
2. Information was presented about the common communication difficulties and the preserved abilities of people with dementia at different stages of dementia. For example, in late and middle stages of dementia, there may be difficulties in initiating conversation, finding the right words in conversation, and talking clearly. General communication strategies to address each of these difficulties were described in a PowerPoint presentation.
3. Video YouTube demonstrations called MESSAGE,^21^ communication in dementia, were viewed. Interactive discussions regarding the effectiveness of the interactions were facilitated by the instructor, and the participants gave suggestions for alternative strategies that could have been used. Care providers also participated in role-playing activities with each other to practice applying the strategies, which included switching roles as care provider and resident. The goal of this activity was to engage workshop participants in applying the strategies and to maximize group interaction with the instructor and each other.
4. Information about and training on individual communication plans was provided. Care providers collaborated with the SLP to modify and enhance the care plans for each resident enrolled in the study. This section was essential to personalize the plans to best fit the residents and their care providers.

#### Supporting implementation of the communication plans

The last component of the RCCI involved the implementation of a support system for care providers as they were carrying out the communication plans. An advanced practice nurse (M.S.) met weekly with care providers to review and assist with the implementation of the communication plan. This strategy aimed to support, mentor, and teach care providers at the bedside by modeling and reinforcing new skills.

### Variables and Measures

#### Sample characteristics

Data were collected from health records on resident characteristics, including age, sex, length of time on the unit, and medical diagnoses. In response to standard questions, the care providers provided data on their age, sex, education, job training, job status, language(s) spoken, and length of employment in the LTCH.

#### Resident baseline measures

Baseline characteristics related to cognition were evaluated using the Mini-Mental State Examination (MMSE).^22^ Functional communication was measured with the Functional Linguistic Communication Inventory (FLCI).^23^ The FLCI consists of 32 items that evaluate 10 components: greeting and naming, question answering, writing, sign comprehension and object to picture matching, word reading and comprehension, ability to reminisce, following commands, pantomime, gesture, and conversation. The FLCI has high test–retest reliability, evidenced by reliability coefficients for the 10 subscales ranging from 0.69 to 0.92.^23^

A number of methods were used to screen hearing and vision depending on the cognitive and linguistic abilities of the residents. Rather than using the Snellen test for visual acuity, the preferred test for persons with dementia is the Teller acuity test because persons with dementia have difficulty concentrating long enough to recognize letters on standard visual acuity charts or communicating clearly enough to identify the targets they see.^24^ Color vision was tested using the Ishihara color vision test.^25^ Hearing was screened by an audiologist. Pure-tone thresholds were tested with a portable audiometer (Grason Stadler 18 with DDR 45 headphones St. Eden, Prairie, MN 55344) in the quietest available room. If the resident was unable to complete pure-tone audiometry, then other measures (eg, live voice testing) were used to obtain a baseline indication of functional ability to hear speech at a typical conversational level. The results and the challenges of collecting these baseline measures have been highlighted in another article.^26^

#### Resident outcomes

Resident outcomes were derived from the following 3 measures: (1) the Cornell Scale for Depression in Dementia^27^ comprising 19 items clustered into 5 domains (mood-related signs, behavioral disturbance, physical signs, cyclical functions, and ideational disturbance), (2) the Alzheimer Disease-Related Quality of Life (ADRQL)^28^ comprising 40 items clustered into 5 domains (social interaction, awareness of self, feelings and mood, enjoyment of activities, and response to surroundings), and (3) the index of independence in ADL (Katz index of ADL)^29^ comprising 6 individual items to detect problems in performing ADLs (ie, bathing, dressing, toileting, transferring, continence, and feeding).

#### Care provider outcomes

Care providers’ outcomes were derived from the following 4 measures: (1) the Communication-Impairment Questionnaire (CIQ)^30^ involving 8 items to measure the attitudes of the care providers about communicating with residents who have communication impairments; (2) the Interactional Comfort Survey^31^ involving 5 domains to measure care providers’ perception of their competence, confidence, willingness, frequency, and scope of practice related to interacting with residents; (3) the Satisfaction Working with Residents with Dementia^32^ scale consisting of 21 items to measure care providers’ satisfaction when working with this population; and (4) a modification of the Nursing Care Assessment Scale (M-NCAS based on the NCAS of Novak and Chappell^33^), involving 28 items incorporated into 2 domains, the care provider’s perception of the residents’ behaviors (behavioral domain) and the care providers’ strain related to each behavior (burden domain).

#### Care provider adherence to the communication plans

Adherence was examined by observing the care provider–resident dyad at time 2. The observation was done on a day when the care provider was assigned to provide care to the participating resident at a time mutually agreed upon by the care provider and resident with no signs of dissent from the resident. The RA observed the interaction between the care provider and resident and documented the performance of the dyad using the Interaction Rating Form (IRF) developed by Shelton and Shryock.^34^ The IRF includes a checklist of verbal and nonverbal strategies that are commonly used when interacting with persons with communication impairments. The IRF provides data on the number of strategies that were specified in the communication plans and delivered during the interaction by the care provider. Higher total IRF scores indicate higher levels of adherence to the strategies suggested in the communication plans. Interrater reliability (*r* = .91-.95) and construct validity (*r* = .84-.96 with clinical judgment) have been reported previously.^34^

#### Care provider perceptions of the RCCI

At time 2, depending on their preferences and the availability of the care providers, focus group or individual interviews were conducted with them to explore their perceptions of experiences with the RCCI. The PI and the RA facilitated the focus group sessions, guided by semi-structured questions and prompts to clarify or elaborate on participants’ responses. The questions addressed participants’ perception of (1) the relevance of the content of the workshop to practice and the effectiveness of the teaching–learning techniques in enhancing care providers’ understanding of the communication strategies and promoting meaningful interactions with residents; (2) the individualized resident communication care plan, in terms of its appropriateness in addressing residents’ communication needs, utility in enhancing care provider–resident interaction, and ease of implementation; and (3) factors that facilitated or hindered interactions with residents, as well as additional benefits of the RCCI.

### Data Analysis and Statistical Methods

The quantitative data were analyzed using STATA version 14.^35^ Descriptive baseline characteristics of participants (ie, residents and care providers) and the rate of adherence to the communication plan were presented as means and standard deviations (SDs) or percentages. Differences in outcomes between time points were analyzed using multilevel mixed-effects linear regression with 2-way crossed random effects (residents and care providers) to account for the repeated measures within dyads, repeated sampling of the same residents, and repeated sampling of the same care providers. Time was a fixed effect, as was each characteristic of care providers and of residents. In examining the care providers’ adherence to the communication plans, the unit of analysis was the interaction of the care provider–resident dyad observed by the RA. The number of communication strategies applied over the observed period was computed for each dyad; these data were analyzed descriptively (mean and frequency) to determine whether care providers used the strategies suggested in the communication plans. To examine care providers’ perceptions of the intervention, written field notes were transcribed following the focus group sessions and the questions acted as a guide for comparative analysis to identify themes.^36^ Two of the authors (R.W. and B.M.B.D.) independently reviewed and coded the field transcripts, reaching an initial consensus of over 96% on the coding. With further discussion, 100% consensus was reached.

## Results

### Description of the Sample

#### Resident sample

A total of 12 residents took part in the intervention (Table 1). The mean age of the residents was 87 years (SD = 5); most were female (83%) and had lived in the LTCH on average for 17 months (SD = 9), with 1 outlier of 108 months (9 years). On average, residents were affected by 3 comorbidities (SD = 1) and were severely cognitive impaired as evidenced by a mean MMSE score of 11/30. They had many functional linguistic communication challenges as indicated by a mean score of 37/82.

**Table 1.** Baseline Resident Demographics.

Characteristics (n = 12)	
Age, mean ± SD (range), years	86.6 ± 5.2 (77-94)
MMSE, mean ± SD (range)	11.2 ± 6.0 (2-20)
FLCI, mean ± SD (range)	37.8 ± 23.0 (6-82)
Sex	
Female, number (percentage)	10 of 12 (83%)
Male, number (percentage)	2 of 12 (17%)
Length of stay, mean ± SD (range), months	17 ± 9 (5-30), excluding outlier of 108 months
Comorbidities, mean ± SD (range)	3 ± 1.4 (1-5)

Abbreviations: FLCI, Functional Linguistic Communication Inventory; MMSE, Mini-Mental State Examination; SD, standard deviation.

#### Care provider sample

A total of 20 care providers consented to participate in the study (Table 2). Their mean age was 45 years (SD = 8). Most were female (75%), had a Health Care Aide Program Certificate (95%), were working full-time (70%), and had worked at least 10 years in the facility (55%; range 1.5-22 years). Most care providers were nonnative English speakers (65%), and 95% had received some type of formal training on communication in dementia care prior to the study.

**Table 2.** Baseline Care Provider Demographics.

Characteristics (n = 20)	
Age, mean ± SD (range), years	45.4 ± 8 (33-60)
Sex	
Female, number (percentage)	15/20 (75%)
Male, number (percentage)	5/20 (25%)
Currently working	
Full time (40 h/wk), number (percentage)	14/20 (70%)
Part time (20 h/wk), number (percentage)	6/20 (30%)
Educational background	
Health Care Aide Certificate, number (percentage)	19/20 (95%)
Bachelor of Nursing, number (percentage)	1/20 (5%)
Years worked in the facility, mean ± SD (range)	10 ± 4 (1.5-22)
Spoken language	
Unilingual (English), number (percentage)	7/20 (35%)
Bilingual, number (percentage)	13/20 (65%)

Abbreviation: SD, standard deviation.

#### Effects of the RCCI on resident outcomes

Several subcomponents of ADRQL improved from time 1 to time 2 (see regression results in Table 3): feelings and mood (*P* = .02), response to surroundings (*P* = .03), and the overall score (*P* = .01), but there was no significant change in residents’ depression (*P* = .80) or ADLs (*P* = .35).

**Table 3.** Comparison of the Mean Scores of Outcome Variables for the Residents and the Care Providers Before and After the Intervention Using Multilevel Mixed-Effects Linear Regression.

	Random-Effect Parameters				Fixed-Effect Parameters		
	SD Resident–Care Provider Dyads	SD Residents	SD Care Providers	SD Residual	Mean at Baseline	Mean 3-Month Postintervention	*P* Value
Resident outcomes (n = 12)							
ADRQL	6	9	8	6	64	70	.01
ADRQL subcomponents							
Response to surroundings	3	16	9	20	60	73	.03
Feelings and mood	12	20	0	10	53	60	.02
Awareness of self	8	15	0	17	58	64	.26
Enjoyment of activities	0	5	25	18	56	60	.42
Social interaction	2	5	19	10	82	84	.51
CSDD	0	0.2	0.2	0.4	0.70	0.67	.80
Index of independence in ADL (Katz index of ADL)	1.2	2.3	0.4	1.0	13.2	12.9	.35
Provider outcomes (n = 20)							
Attitudes and Behaviors: Modified Nursing Care Assessment Scale	0.1	0.2	0	0.2	2.08	2.34	.001
Care Providers’ Burden or Strain: Modified Nursing Care Assessment Scale	0.2	0	0.2	0.3	2.33	2.11	.03
CIQ	0.2	0	0	0.5	3.8	3.5	.10
PICS	0.1	0	1.1	1.0	8.0	8.4	.23
SWRD	7.3	0	0	5.8	5.9	6.1	.27

Abbreviations: ADL, activities of daily living; ADRQL, Alzheimer’s Disease-Related Quality of Life; CIQ, Communication-Impairment Questionnaire; CSDD, Cornell Scale for Depression in Dementia; PICS, Providers Interactional Comfort Survey; SD, standard deviation; SWRD, satisfaction working with residents with dementia.

#### Effects of the RCCI on care provider outcomes

The care providers’ attitudes toward residents with communication impairment (ie, total CIQ scores) were compared between time 1 and time 2 using multilevel mixed-effects linear regression (Table 3); the difference did not reach statistical significance (*P =* .10). However, care providers’ scores increased significantly on the behaviors (feelings/moods) subscale (*P* = .001) and decreased significantly on the burden or strain subscale (*P =* .03; Table 3).

#### Care providers’ adherence to the communication plan

Analysis of the observation data showed that 11 care providers had 100% adherence; they implemented all suggested communication strategies whenever appropriate as outlined in the resident’s tailored communication plan. Five care providers carried out 80% to 92% of the strategies, whereas 2 care providers performed 40% to 55% of the strategies. Two care providers withdrew before time 2 data collection. Overall, the mean adherence rate was 91%.

#### Care providers’ perceptions of the intervention

A total of 12 care providers shared their perception on the RCCI; 5 attended a focus group and 7 were interviewed individually. Several themes were identified, and an objective description was developed for every theme. Refinements were conducted until a stable set of concepts was related to the theme under study. Major themes included the utility of the communication plan, the influence of the plan on the residents’ well-being, and barriers and facilitators to using the communication strategies.

Analysis of data revealed that, overall, care providers viewed the RCCI positively. Participants commonly reported that it promoted resident-centered dementia care, which included understanding the person’s preferences, the dementia process, and the meaning of behaviors, and how to effectively respond to behaviors during care.

#### Communication plan

Care providers indicated that the plan was a helpful tool because it was easy to read, the different sections were relevant, and it was easily accessible (eg, the location where the care plan was kept was convenient). Additionally, care providers identified several ways that the care plan had a positive effect on their practice, including improved individualized resident care, improved (verbal and nonverbal) communication with residents, and increased confidence when providing care. For example, 1 care provider said “the care plan boosted my confidence with the resident and my ability to express myself.”

The plan also acted as a support for care providers, such as serving as a reminder for action (eg, “It helped, it opened more doors, although, sometimes we don’t do what we’re supposed to and it acts as a reminder”). The care providers also indicated that the intervention could help part-time care providers to learn about residents who were unfamiliar to them. Importantly, care providers indicated that if part-time staff had access to this resource, instead of asking the full-time staff questions, they would have more time for resident care.

#### Residents’ well-being

Overall, care providers noted the positive effect of the individualized care plan on the residents’ QOL and self-efficacy as it met their individualized needs and reduced responsive behaviors. For example, 1 care provider commented: “It helps, especially with the care, because it gives you a way to properly approach the resident and when to approach them. It also helps to plan care based on their needs.” Another caregiver provided an example: “…at bedtime, with my resident, I provide choices on what she would like to wear to bed, which makes her happy because it is her own choice and allows her to be independent.”

#### Barriers to using communication strategies with residents

After highlighting the positive aspects of the intervention, caregivers were candid and reported on the barriers. The main concern was related to the limited time that care providers have for interpersonal communication with residents due to their workload and insufficient staffing. Others expressed their views that a lack of consistency between coworkers’ approaches to care may be a barrier to communicating with residents. Notably, 1 care provider was worried that the intervention strategies may not coincide with care provider safety during care (eg, “Sometimes I am not comfortable with the suggested interventions, like getting close, you don’t want to get hit.”).

#### Facilitators to using communication strategies with residents

Care providers highlighted the need to maintain consistent use of the communication plan among all care providers in order to facilitate the use of the individualized communication strategies with residents. Also, the care providers indicated the importance of the culture of the workplace in terms of supporting the use of time to communicate (eg, “The work culture helps. We don’t rush the residents on the floor.”).

## Discussion

Current evidence suggests that delivering individually tailored resident interventions may improve the QOL of residents with dementia.^10^ In the present study, the investigators attempted to operationalize resident-centered practices by developing individualized communication plans focused on the needs of individual residents living in LTCH; these needs were identified during screening assessments across multiple domains related to communication and with input from care providers. Adherence to the communication plans was high; on average, care providers carried out 91% of the strategies suggested in the plans. While depression and ADL showed no significant change after the RCCI, there was an overall improvement in resident QOL postintervention. The findings of the present study are consistent with previous findings from similar work^11^; general QOL has been shown to be most responsive to communication intervention in previous studies. Furthermore, care providers scored significantly higher on postintervention measures of feelings and mood, while their scores on the burden and strain subscales decreased similar to findings by other investigators.^14^ It would appear that we can help care providers communicate more effectively with persons having dementia in LTCH and also make caregiving easier. The present findings of positive influences on care provider outcomes as a result of using individualized communication plans are consistent with previous findings of similar programs for persons recovering from stroke.^16^

Several areas for improvement were identified, including those based on feedback from care providers during focus groups, observations during screening testing, insights gained during the development of the communication plans, and ongoing evaluation of the implementation of the communication plans in context. Notably, the care providers felt the plans were useful and helped them to truly understand some important nuances when interacting with the residents. Specifically, care providers felt that the plans helped with (1) learning about residents on a personal level, (2) behavior management, and (3) improving care planning by providing information on when and how to approach residents based on their needs and preferences. Care providers liked the fact that the care plans were short, easy to read, presented in point form, and tailored to the individual resident. Further refinement of the care plans should include more information on what is required when the resident has vision or hearing challenges (or both). Incorporating feedback from staff in future larger trials will be critical for successful implementation of the intervention.

After the workshop, the SLP incorporated feedback from the care providers into the care plans. This input was particularly important for some residents who had behavioral challenges. For example, 1 resident had very limited communication, which was primarily nonverbal. The care provider suggested that her care should be left until last, because she became aggressive if she was woken up early and she did not like being rushed. She also noted that calling her “Miss Canada” and complimenting her appearance was helpful, and listening to music on her personal iPod helped to calm her down. These insights could only be based on care providers’ observations of the resident in context.

Although sensory and linguistic assessments provide important information about the abilities of residents, more information about the activities and typical communication environments of residents is needed to determine which specific strategies are useful for enhancing communication function in the daily situations relevant to individual residents. It was therefore important for the SLP, advance practice nurse, and experts in cognition, vision, and hearing to meet with each resident at the LTCH, so that the care plan could be refined to best fit the individual resident in context. Thus, care plans should then be modified based on ongoing monitoring of the quality of the interactions between care providers and residents.

### Limitations of Study

A few limitations of the present study are noted. This study design did not include a control group, which may influence the internal validity of the findings concerning the effectiveness of the intervention. However, finding equivalent resident controls in nursing home research is not easily achieved given the heterogeneity of this population. Another limitation is that the care plans were detailed and developed based on input from a number of experts who are not typically available as resources in most LTCH settings. Educating staff, hiring health professionals such as SLPs to conduct assessments, and facilitating knowledge exchange at the bedside are resource intensive, and more reflection from administrators is required to understand how this can best be achieved. However, the costs of the intervention might be mitigated by reducing staff turnover over time, if staff feel less burdened following the intervention. Furthermore, assessing the sensory abilities of persons with moderate-to-severe cognitive impairment required the use of nonstandard testing methods that were adapted according to the needs of the individual residents and situation. A new study to determine the most feasible and effective methods to assess the sensory abilities of residents in LTCH is underway so that the information from sensory assessments can be used to improve care plans and, in turn, the interactions between care providers and residents in LTCHs.^26^ Finally, although only 1 observation was made between the care provider and resident, care providers affirmed that these were typical interactions.

## Conclusion

The number of persons with dementia living in nursing homes continues to grow. A resident-centered intervention involving the development of communication plans based on the abilities of residents with dementia can support care providers in practice and have beneficial effects on their feelings and mood, while reducing their burden. This approach can also have beneficial effects on residents’ QOL. The findings of the present study contribute to a growing body of evidence that individualized resident-centered interventions improve resident and care provider outcomes.
